# Clozapine-Induced Cardiotoxicity Presenting as Sepsis: A Case Report and Literature Review

**DOI:** 10.1155/2019/3435108

**Published:** 2019-03-31

**Authors:** Mazin Khalid, Oluwole Jegede, Vijay Gayam, Ying Chi Yang, Binav Shrestha, Amrendra Mandal, Osama Mukhtar, Pavani Garlapati, Mowyad Khalid, Alix Dufresne

**Affiliations:** ^1^Department of Medicine, Interfaith Medical Center, Brooklyn, NY, USA; ^2^Department of Psychiatry, Interfaith Medical Center, Brooklyn, NY, USA; ^3^Department of Medicine, Detroit Medical Center, Wayne State University, Detroit, MI, USA; ^4^Department of Cardiology, Interfaith Medical Centre, Brooklyn, NY, USA

## Abstract

Clozapine is an atypical antipsychotic agent indicated in the treatment of medication-resistant schizophrenia. It is often reserved as a last line of treatment owing to the potential for serious and potentially life-threatening side effects, the most serious being agranulocytosis requiring close hematological monitoring and possible discontinuation of the medication from further use in the patient even when the agranulocytosis resolves. Other complications of clozapine include sedation, weight gain, elevated triglyceride levels, postural hypotension, and tachycardia. However, the potentially serious complication of myocarditis, though rare (with an incidence of 3%), may lead to cardiomyopathy as described in our present case. We present a 21-year-old patient who was started on clozapine for management of schizophrenia. He developed fever and tachycardia and was admitted to the medical unit on intravenous antibiotics for management of sepsis as he met the criteria for systemic inflammatory response syndrome. His labs revealed an elevated troponin and trending eosinophilia, which, in the context of clozapine use, raises the suspicion of clozapine cardiotoxicity. Echocardiogram showed reduced systolic function (45%). Clozapine was immediately discontinued, and his repeat echocardiogram showed normalization of his systolic function. In view of the increased prevalence of psychiatric illnesses, internists should be aware of rare but potentially life-threatening side effects.

## 1. Introduction

Clozapine is an atypical antipsychotic agent indicated in the treatment of schizophrenia or schizoaffective disorder patients who are resistant to treatment with other antipsychotics as well as the drug of choice among schizophrenic patients with suicidal ideation. The mechanism of action of clozapine is thought to be mediated through dopamine D_2_ and serotonin 2A receptor antagonism as well as antagonistic actions at alpha-adrenergic, histamine H_1_, and cholinergic receptors. Its wide range of receptor profile also accounts for its attendant side effects [1, 2].

As noted above, clozapine is often reserved as a last-line treatment owing to the potential for serious and potentially life-threatening side effects as such physicians are required to abide by strict prescription guidelines as outlined in the clozapine Risk Evaluation and Mitigation Strategy (REMS) [3]. Clozapine is associated with severe neutropenia (absolute neutrophil count (ANC) less than 500/*μ*L), for which patients have ongoing hematological monitoring. Intestinal obstruction from severe constipation is another cause of mortality for which patients require monitoring and preemptive management. Other side effects are sedation, weight gain, elevated triglyceride levels, postural hypotension, and tachycardia [4–6].

Myocarditis is a rare but potentially serious side effect of clozapine. It has a reported incidence of 3% and may lead to cardiomyopathy [7]. Clozapine-induced pericarditis has also been reported and should be considered as a differential as in a recent case described by Berardis and colleagues in 2018 of a 27-year-old woman with schizoaffective disorder, who developed a sudden onset clozapine-related pericarditis during medication titration [8]. Our present case is that of a young African American man who was transferred from an inpatient psychiatric unit due to suspected sepsis as he met the systemic inflammatory response syndrome (SIRS) criteria and treated accordingly with antibiotics but a closer review of an increasing eosinophilia, a high troponin, the context of his current clozapine regimen, and absence of other clinical explanation of his symptomatology prompted a revision of the diagnosis to myocarditis probably induced by clozapine. Echocardiography revealed a decreased left ventricular ejection fraction (EF) which responded to the withdrawal of clozapine, resulting in the normalization of his EF.

## 2. Case Presentation

The patient is a 21-year-old African American man with a past psychiatric history of schizophrenia who was transferred from the psychiatry unit to the medical floor to rule out sepsis after the development of fever and tachycardia. The patient was reported to have decompensated on account of nonadherence with his home medications resulting in frequent hospitalizations and poor functioning. As per the patient's admission records, the patient's initial labs and urine toxicology were within normal limits. The patient had been on various antipsychotics since his diagnosis with schizophrenia at the age of 19, including haloperidol, olanzapine, and risperidone, but he continued to decompensate despite adequate medication trials. The decision was made to start the patient on clozapine due to possible failure of these previous antipsychotics. Clozapine was started at 25 mg PO twice daily and titrated up to 150 mg PO twice daily over the next 12 days. His current daily dose was 300 mg when he was transferred to the telemetry unit following a sudden development of fever and tachycardia on the psychiatric inpatient floor.

On the medical inpatient unit, the patient was mostly selectively mute and did not appear to be in any pain, was not vomiting, and had no diarrhea and no reported loss of consciousness or seizures. His admission vitals were a temperature of 102.4 degrees Fahrenheit, BP of 115/81 mmHg, HR of 114 beats per minute, and an oxygen saturation of 97% on room air. Physical examination did not reveal any significant findings other than a mild dehydration, no skin rash, no jugular venous distension, basal crepitation or pedal edema. Cardiac auscultative findings were largely normal except for a tachycardic heart rate; apex beat was in the 5th intercostal space, midclavicular line. The patient's heart sounds, S1 and S2, were normal, no rubs, or murmurs, and no obvious gallop rhythm. His peripheral extremities were warm with a normal capillary refill.

**Figure 1:**
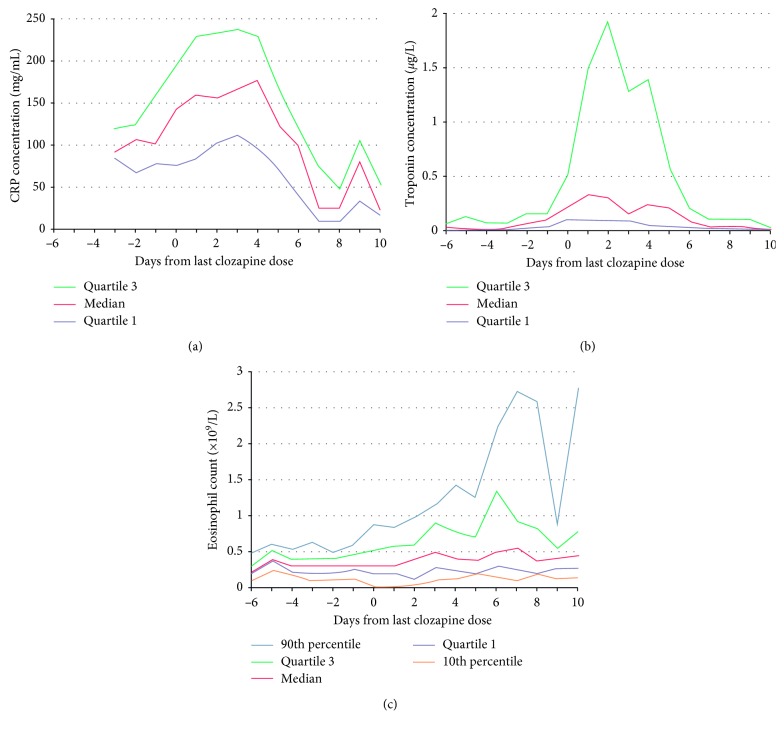
The evolution of (a) C-reactive protein (CRP), (b) troponin I/T, and (c) eosinophil count with time in cases, using the day of the last dose of clozapine (stop date for myocarditis) as the reference. The precipitous drop in the 90th percentile for eosinophil count on day 9 arises from the lack of date from cases with high counts on that day. Descriptive statistics (mean ± standard deviation, range) for the number of results available for each day were CRP 21 ± 14, 5–48; troponin I/T 29 ± 23, 7–83; and eosinophil (Ronaldson et al. [25]).

The patient was given an intravenous bolus of normal saline and a stat dose of intravenous antibiotics (vancomycin and meropenem), and full workup including 2 sets of blood cultures, urine analysis, and a chest radiograph was ordered. His complete blood count (CBC) revealed a normal leukocyte count of 7000/*µ*l with elevated eosinophils of 500 (reference 0.0–400, see Figure 1 for trend) and normal platelet count. He had an elevated erythrocyte sedimentation rate (ESR) of 26 (reference 0–15 mm/Hour) and CRP was 147.4 mg/dl (reference 0.0–4.9 mg/dl).

**Figure 2:**
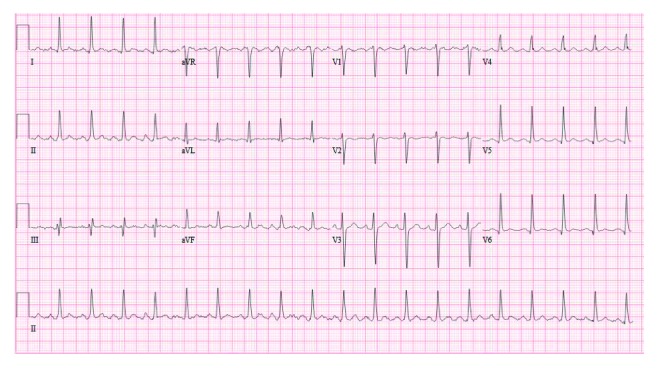
EKG showing sinus tachycardia without ST-T changes.

In view of the patient's current clozapine use, a presumptive diagnosis of myocarditis was made and troponin I was requested which turns out to be markedly increased, 1.12 ng/dl (reference: 0.00–0.05 ng/dl). His NT-pro B-type natriuretic peptide (BNP), lactic acid, and creatine kinase levels were normal. His electrocardiogram (EKG) showed sinus tachycardia without specific ST-T changes (Figure 2 below). His chest radiograph was unremarkable. Urinalysis and urine toxicology were negative. Initial transthoracic echocardiogram (TTE) revealed a left ventricular systolic dysfunction with apical hypokinesis (ejection fraction 45%) and mild tricuspid regurgitation. Coronary angiogram showed patent coronaries; ventilation/perfusion scan resulted as low probability for pulmonary embolism.

Clozapine was discontinued on admission to the medical unit. Subsequently, the patient's fever and tachycardia resolved. Troponin I trended down (lowest being 0.22 ng/ml). Eosinophil count initially increased but later normalized as shown in Figure 1. Repeat transthoracic echo after one week of discontinuing clozapine revealed normal left ventricular systolic function with mild apical hypokinesis (ejection fraction of 60–65%). The patient was clinically stable throughout the hospital course. The psychiatry consult liaison team started the patient on aripiprazole which was well tolerated by the patient without any side effects. The patient later underwent an uneventful discharge to the community outpatient clinic.

## 3. Discussion

Clozapine myocarditis has an incidence of 3% and mostly occurs in the first 2-3 months after starting treatment [9]. The mechanism of clozapine myocarditis has not been established in the literature, but IgE-mediated hypersensitivity, genetic predisposition, and cholinergic dysfunction have been proposed as possible causes [10]. The common presenting symptoms are shortness of breath, palpitations, cough, and fatigue [8]. Kilian et al. reviewed 8000 patients on clozapine and reported 23 cases of myocarditis and cardiomyopathy and cited fever in association with chest pain or dyspnea as a presenting symptom, but fever as a sole presenting symptom has not been reported [12]. These variable presentations are prevalent without cardiac involvement, especially early in the treatment course of clozapine [7].

Berardis et al. conducted a literature review and summarized the common presenting symptoms of clozapine myocarditis as shown in Table 1. They also reported that polypharmacy with other agents (i.e., valproate and aripiprazole) may increase the risk of clozapine-induced myocarditis and pericarditis [3]. Ronaldson et al. conducted a literature review and reported that fever may develop prior to the cardiac insult and can result in missing the diagnosis as fever may represent a general side effect of clozapine [7]. As is the case with our patient, laboratory testing usually reveals a progressive increase in peripheral eosinophil counts as well as increase in troponin, creatine kinase-MB, and BNP levels (see Figure 1 for eosinophil count trend). Electrocardiogram usually shows nonspecific findings [7, 11, 13]. Echocardiography (Echo) is a helpful tool in assessing the systolic cardiac function, namely, the left ventricular ejection fraction (LVEF) [7, 10, 14, 15]. Although endomyocardial biopsy is the modality for definitive diagnosis, this is usually avoided due to the risks associated with the procedure and the possibility of missing focal myocarditis [15, 16]. The presence of suggestive clinical findings, echo findings, and an improvement is usually sufficient for diagnosis, especially if supported by the resolution of symptoms following the withdrawal of the medication [9].

Stress cardiomyopathy is a less likely possibility since the patient was catatonic and in a psychiatry facility for almost two weeks prior to the presentation without remarkable stress. In addition, the presence of high-grade fever and the resolution after the withdrawal of the offending agent further supports the diagnosis. Another entity to be considered in this case is eosinophilic myocarditis (EM). EM is divided classically into three chronological stages: eosinophilic infiltration, thrombosis, and fibrosis [17]. Patients with EM may present with various symptoms ranging from palpitations, fever, chest pain, and shortness of breath to heart failure and cardiogenic shock [18, 19]. The gold standard noninvasive modality for diagnosis of EM is cardiac magnetic resonance imaging (MRI); however, it is not widely available and costly. Endomyocardial biopsy remains the best modality to reach a definitive diagnosis [20]. Steroids are the standard treatment for EM, but there is no clear consensus on the dosage. According to Ogbogu et al. in a retrospective study, 85% of the patients experienced complete or partial response after 1 month of treatment [21].

Our patient's clinical picture, in addition to the laboratory findings, supported the diagnosis of clozapine myocarditis which was further confirmed by the echocardiographic finding of decreased LVEF and the dramatic improvement of the LV function following the withdrawal of the offending agent. Cardiac catheterization was done and did not reveal any obstruction. While clozapine myocarditis has been attributed to IgE-mediated hypersensitivity, the presence of peripheral eosinophils has been well documented, an unmistakable upward trend clearly identified in our case.

The management of clozapine myocarditis starts with the prompt discontinuation of the causative agent. Standard heart failure treatment has been used for myocardial function support. A combination of angiotensin-converting enzyme inhibitor (ACE), diuretic, and beta blockers are commonly used medications for treatment. Subjectively, normalization of the heart rate and improvement of the heart failure symptoms can be a good clinical indicator to document recovery. An objective method of documenting recovery is by trending inflammatory markers such as CRP and ESR and repeating echo [9, 10]. Re-challenging patients with clozapine after myocarditis remains unclear but usually not advised. Clozapine remains the most effective medication in refractory schizophrenia; therefore, re-challenging has been attempted in some cases [22].

Monitoring clozapine cardiac side effects have been challenging, and thus far, there is no consensus on a monitoring approach. Ronaldson et al. proposed a myocarditis screening protocol for all patients beginning treatment with clozapine. This proposed monitoring protocol recommends obtaining baseline troponin I/T, C-reactive protein, and echocardiography and monitoring troponin and C-reactive protein on days 7, 14, 21, and 28. They concluded that monitoring should not be conditional on the presence of clinical symptoms, but it should be intensified in those who develop a physical illness within 4 weeks of commencing clozapine. Those with laboratory results suggestive of myocarditis should have clozapine discontinued, to prevent further injury [23]. After publication, it was criticized for not being cost-effective and, if made mandatory, for possibly introducing a barrier to initiating clozapine therapy in areas with limited resources [24]. In 2015, the same group wrote an article with graphical display of the timing of various markers elevation in relation to clozapine [25]. While patients are on clozapine, a high index of suspicion should be maintained with regards to—serious but rare—cardiac adverse reactions. Physicians should employee their physical examination and laboratory testing such as ESR, CRP, and troponin towards early detection of adverse cardiac reactions on a case to case, especially in the first 4 weeks of treatment. Further testing and follow-up should be decided on a case-to-case basis.

## 4. Conclusion

In view of the increased prevalence of psychiatric illnesses, internists should be aware of rare but potentially life-threatening side effects of psychotropic medications. The early detection and management of sepsis is important in the reduction of morbidity and mortality. In our patient, at initial evaluation, his symptoms appeared to be due to sepsis but an in-depth review of the case revealed a potentially dangerous adverse drug reaction. This case highlights clozapine-induced myocarditis, which is a rare but serious side effect. Physicians should pay attention to the subtle changes in laboratory results to a review of the potential adverse effects of psychiatric medications.

## Figures and Tables

**Figure 3:**
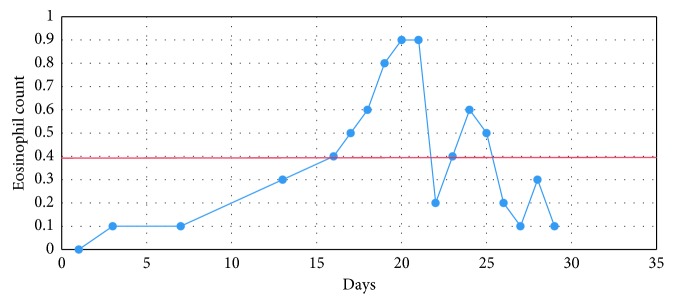
Patient's eosinophil trend in the course of hospitalization.

**Table 1:** Common symptoms associated with clozapine myocarditis [3].

More common	Less common
Flu-like symptoms such as fever, myalgia, dizziness or faintness, arthralgia, nasal congestion, and sensations of “scratchy throat”	Dysuria
Fatigue or decreased exercise tolerance	Left shoulder pain on inspiration
Respiratory symptoms such as dyspnea, cough, subjective sensation of chest discomfort, and orthopnea	Rash
Cardiovascular symptoms such as persistent resting tachycardia, increased heart rate, palpitations, chest pain, syncope, arrhythmia, and hypotension	Cyanosis
Acute mental status change and delirium	Seizures
	Dysarthria
	Peripheral edema
	Paroxysmal nocturnal dyspnea
